# Identification of lactate dehydrogenase as a mammalian pyrroloquinoline quinone (PQQ)-binding protein

**DOI:** 10.1038/srep26723

**Published:** 2016-05-27

**Authors:** Mitsugu Akagawa, Kenji Minematsu, Takahiro Shibata, Tatsuhiko Kondo, Takeshi Ishii, Koji Uchida

**Affiliations:** 1 Department of Biological Chemistry, Division of Applied Life Science, Graduate School of Life and Environmental Sciences, Osaka Prefecture University, Sakai 599-8531, Japan; 2 Graduate School of Bioagricultural Sciences, Nagoya University, Nagoya 464-8601, Japan; 3 PRESTO, Japan Science and Technology Agency (JST), Kawaguchi, Saitama 332-0012, Japan; 4 Faculty of Nutrition, Kobe Gakuin University, Kobe, Hyogo 651-8586, Japan

## Abstract

Pyrroloquinoline quinone (PQQ), a redox-active *o*-quinone, is an important nutrient involved in numerous physiological and biochemical processes in mammals. Despite such beneficial functions, the underlying molecular mechanisms remain to be established. In the present study, using PQQ-immobilized Sepharose beads as a probe, we examined the presence of protein(s) that are capable of binding PQQ in mouse NIH/3T3 fibroblasts and identified five cellular proteins, including l-lactate dehydrogenase (LDH) A chain, as potential mammalian PQQ-binding proteins. *In vitro* studies using a purified rabbit muscle LDH show that PQQ inhibits the formation of lactate from pyruvate in the presence of NADH (forward reaction), whereas it enhances the conversion of lactate to pyruvate in the presence of NAD^+^ (reverse reaction). The molecular mechanism underlying PQQ-mediated regulation of LDH activity is attributed to the oxidation of NADH to NAD^+^ by PQQ. Indeed, the PQQ-bound LDH oxidizes NADH, generating NAD^+^, and significantly catalyzes the conversion of lactate to pyruvate. Furthermore, PQQ attenuates cellular lactate release and increases intracellular ATP levels in the NIH/3T3 fibroblasts. Our results suggest that PQQ, modulating LDH activity to facilitate pyruvate formation through its redox-cycling activity, may be involved in the enhanced energy production *via* mitochondrial TCA cycle and oxidative phosphorylation.

Pyrroloquinoline quinone (PQQ), a redox-active *o*-quinone, is an important nutrient involved in a multitude of physiological and biochemical processes in both bacteria and higher organisms^1^,^2^,^3^. Although PQQ has been demonstrated to act as a redox cofactor of bacterial dehydrogenases, such as alcohol and sugar dehydrogenases^4^, its role as a mammalian enzyme cofactor has not yet been elucidated. PQQ is not biosynthesized in eukaryotic organisms, including mammals. However, trace amounts of PQQ can be detected in human and rat organs or tissues^5^ because of its presence in daily foods, such as vegetables and meats, at pM to nM levels^6^,^7^. Most importantly, nutritional studies on rodent models have demonstrated that PQQ deprivation displays divergent systemic responses, such as growth impairment, compromised immune responsiveness, abnormal reproductive performance, and reduced respiratory quotient^8^,^9^. Kasahara and Kato previously identified U26 as a potential PQQ-dependent enzyme, containing a putative PQQ-binding motif, in mice and observed that the enzyme could be involved in the metabolic degradation of dietary lysine, acting as a PQQ-dependent 2-aminoadipic 6-semialdehyde dehydrogenase (AASDH)^10^. Because all bacterial PQQ-dependent dehydrogenases reported to date have a characteristic consensus structure, PQQ-binding β-propeller motif, for PQQ-dependent proteins^4^,^11^,^12^, they concluded PQQ to be a newcomer to the B group of vitamins. However, the claim for a mammalian vitamin was subsequently questioned by other scientists because no PQQ-dependent AASDH activity was detected in mammalian tissues either *in vivo* or *in vitro*, and U26-dependent oxidation of 2-aminoadipate semialdehyde to 2-aminoadipate has never been experimentally demonstrated^13^,^14^,^15^. In addition, Drozak *et al*. recently showed that U26 is a β-alanine-activating enzyme, which catalyzes β-alanine transfer onto thiols in a PQQ-independent manner^16^. Thus, PQQ is not currently accepted as a vitamin.

On the other hand, pyranose dehydrogenase was recently identified as a novel eukaryotic PQQ-dependent enzyme from *Coprinopsis cinerea*^17^,^18^. This enzyme has low homology with the alignment of the amino acid sequence contributing to the binding of PQQ to the enzymes. However, it tightly binds PQQ and exhibits PQQ-dependent enzyme activity. These findings suggest that there is a diversity of PQQ-binding motifs and the possible existence of an unknown PQQ-dependent enzyme. In the present study, to identify a mammalian PQQ-dependent enzyme, we attempted to purify PQQ-binding proteins in mouse NIH/3T3 fibroblasts using PQQ-conjugated Sepharose (PQQ-Sepharose) beads as an affinity probe and identified several enzymes, including l-lactate dehydrogenase (LDH). Based on the identification of LDH as a mammalian PQQ-binding enzyme, we kinetically characterized the effects of PQQ and its reduced form, pyrroloquinoline quinol (PQQH_2_), on the enzymatic reaction of LDH. Although neither PQQ nor PQQH_2_ functioned as the cofactor for LDH, we unexpectedly found that PQQ significantly enhances pyruvate production and inhibits lactate production by LDH in the presence of NADH or NAD^+^. Based on these findings, we propose a novel mechanism, in which PQQ-bound LDH is involved in the conversion of lactate to pyruvate. Moreover, we also show that the exposure of NIH/3T3 fibroblasts to PQQ causes reduced accumulation of lactate and elicits enhanced ATP production.

## Results

### Identification of LDH-A as PQQ-binding target

**Figure 1:**
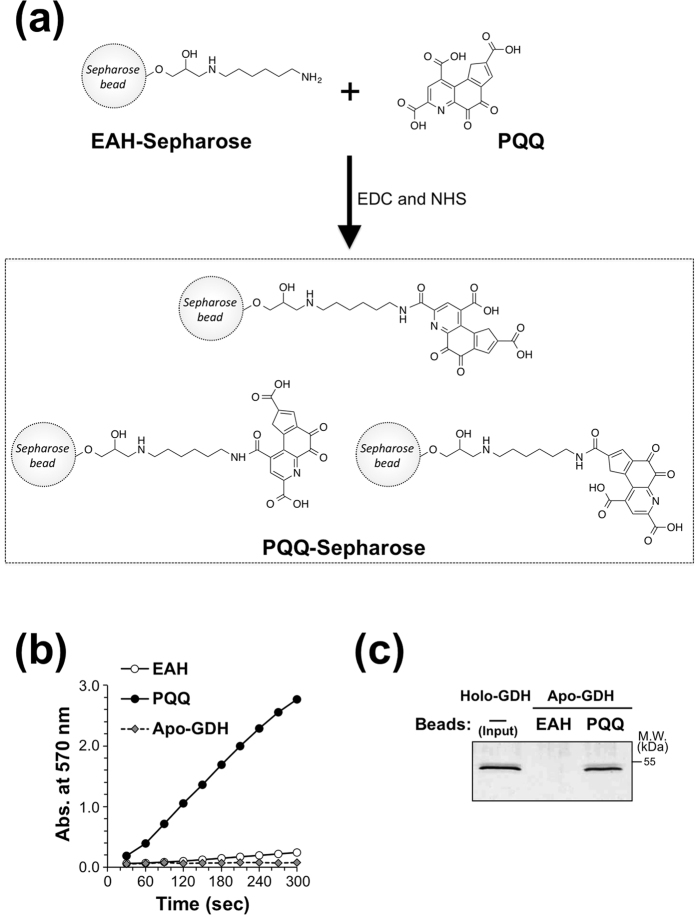
Evaluation of the cofactor activity and binding affinity of PQQ-Sepharose beads to apo-GDH. (**a**) Scheme of PQQ immobilization to EAH-Sepharose beads. Details are described in the Experimental Procedures section. (**b**) Activation of apo-GDH in the presence of PQQ-Sepharose beads. Apo-GDH was incubated with vehicle (Apo-GDH), EAH-Sepharose (EAH), or PQQ-Sepharose (PQQ) beads for 30 min at room temperature. Then, GDH activity was determined from the formation of diformazan by the reduction of NTB with PMS in the presence of glucose. The time course of diformazan formation was measured at 570 nm using a microplate reader. (**c**) Specific binding of apo-GDH to PQQ-Sepharose beads. Apo-GDH was incubated with EAH-Sepharose (EAH) or PQQ-Sepharose (PQQ) beads for 1 h at room temperature. After washing the beads, bound protein was eluted with free PQQ. The input (holo-GDH) and each eluate were analyzed by SDS-PAGE followed by silver staining.

To identify a mammalian PQQ-binding protein, we developed an affinity pull-down assay using the PQQ-Sepharose beads. PQQ was covalently immobilized on the amine-modified Sepharose (EAH-Sepharose) beads through an EDC coupling reaction (Fig. 1a). To validate the utility of the beads, we determined whether PQQ immobilized on the Sepharose beads maintains its cofactor activity. To this end, apo-GDH, which can be reactivated by PQQ, was incubated with the PQQ-Sepharose beads or EAH-Sepharose beads (control) for 30 min and GDH activity was measured by a spectrophotometric assay. As shown in Fig. 1b, the PQQ-Sepharose beads, but not EAH-Sepharose beads, reactivated GDH. We also confirmed that the PQQ-Sepharose beads could allow purification of GDH (Fig. 1c), indicating the utility of the beads for affinity purification of PQQ-dependent enzymes.

**Figure 2:**
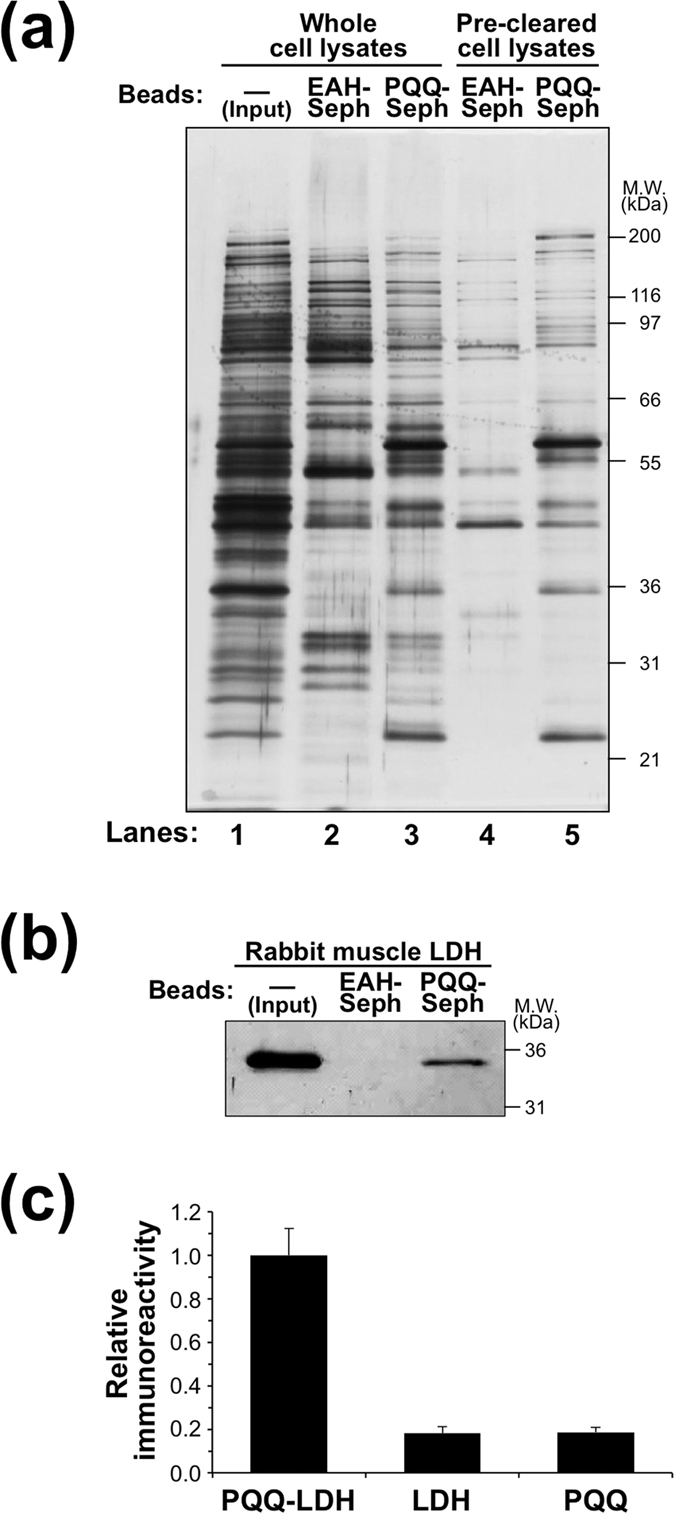
Identification of LDH-A as a PQQ-binding protein. (**a**) Affinity purification of PQQ-binding proteins from NIH/3T3 cells. Whole cell lysates or pre-cleared cell lysates with EAH-Sepharose beads were incubated with EAH-Sepharose (EAH) or PQQ-Sepharose (PQQ) beads for 2 h at room temperature. After washing the beads, bound proteins were eluted with free PQQ. The input (whole cell lysates) and each eluate were analyzed by SDS-PAGE followed by silver staining. (**b**) Binding of rabbit muscle LDH to PQQ-Sepharose beads. LDH from rabbit muscle was incubated with EAH-Sepharose (EAH) or PQQ-Sepharose (PQQ) beads for 1 h at room temperature. After washing the beads, bound protein was eluted with free PQQ. The input (LDH) and each eluate were analyzed by SDS-PAGE followed by silver staining. (**c**) Evaluation of PQQ binding to rabbit muscle LDH by ELISA. Rabbit muscle LDH (100 μg/mL) was incubated with or without 1 mM PQQ in a 96-well polystyrene ELISA plate at 37 °C for 3 h. After washing the plate, PQQ binding was determined by ELISA using anti-PQQ antibody as described in the Experimental Procedures section. The results shown are means ± SE (*n* = 3).

Using the PQQ-Sepharose beads, we sought to identify a mammalian PQQ-binding protein. The NIH/3T3 whole cell lysates were incubated with the PQQ-Sepharose or EAH-Sepharose beads, and the PQQ eluates from the affinity beads were subjected to SDS-PAGE/silver staining. However, high levels of non-specifically bound proteins were detected in both eluates (Fig. 2a, lane 2 and 3). To reduce non-specific binding, the cell lysates were pretreated with the EAH-Sepharose beads and then incubated with the PQQ-Sepharose or EAH-Sepharose beads. The proteins bound to these affinity beads were eluted with PQQ and then analyzed by SDS-PAGE/silver staining. As shown in Fig. 2a, several proteins that specifically bound to PQQ-Sepharose beads were detected. They were then subjected to tryptic digestion followed by nano-LC-ESI-Q-TOF-MS/MS analysis. Tables 1 and 2 summarize the proteins identified in the eluates from EAH-Sepharose and PQQ-Sepharose beads, respectively. Six proteins, including pyruvate kinase PKM, nucleoside diphosphate kinase B, l-lactate dehydrogenase A chain (LDH-A), peroxiredoxin-1, triosephosphate isomerase, and translation elongation factor, were putatively identified as mammalian PQQ-binding proteins.

LDH, among these enzymes, is most likely regulated by PQQ, based on the fact that PQQ-dependent LDHs have already been identified from *Gluconobacter*^19^. Hence, in the present study, we focused on LDH-A (muscle subunit), which is highly conserved in mammals and localized primarily in the cytoplasm of muscle cells. To confirm the binding of LDH-A to the PQQ-Sepharose beads, we carried out a binding assay using a purified rabbit muscle LDH, which is 94% homologous with mouse and human LDH-A (Fig. S1)^20^. The results showed that LDH specifically bound to the PQQ-Sepharose beads (Fig. 2b). We also demonstrated the binding of PQQ to the rabbit muscle LDH by ELISA using an anti-PQQ antibody (Fig. 2c).

### Effect of PQQ on the LDH activity

**Figure 3:**
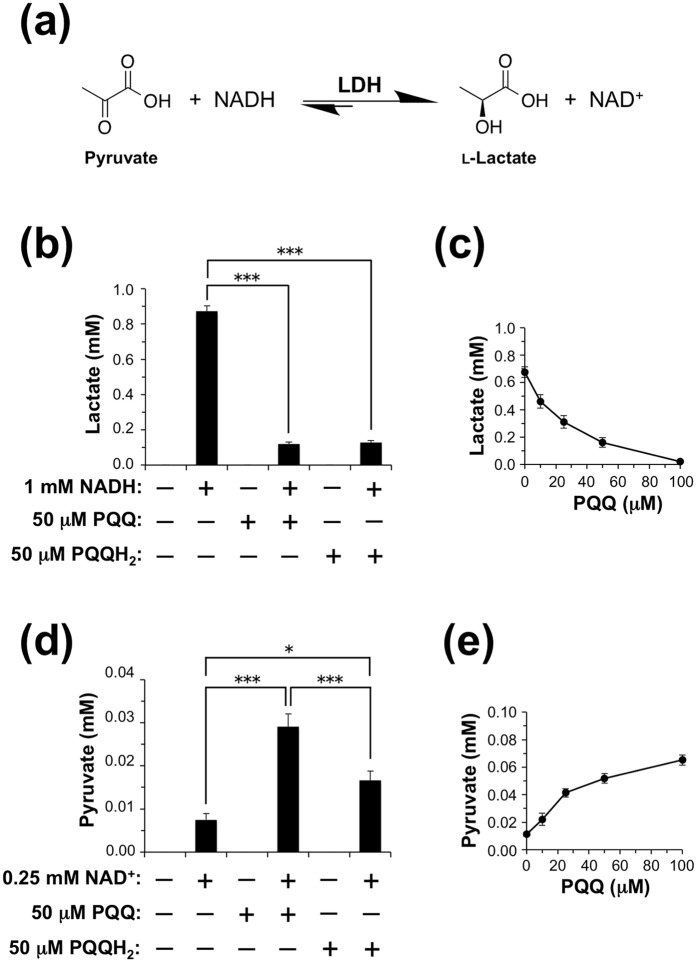
Regulation of LDH activity by PQQ. (**a**) Enzymatic reaction of LDH. (**b**) Effects of PQQ and PQQH_2_ on LDH activity in the forward reaction (pyruvate + NADH → lactate + NAD^+^). Rabbit muscle LDH (0.06 nM) and pyruvate (10 mM) were incubated with or without 1 mM NADH in the presence or absence of 50 μM PQQ or PQQH_2_ at 37 °C for 3 h, and then lactate production was determined by HPLC. The results shown are means ± SE (*n* = 3). *P < 0.05, **P < 0.01, ***P < 0.001 compared as indicated (ANOVA, Tukey-Kramer test). (**c**) PQQ-dependent inhibition of forward reaction of LDH. Rabbit muscle LDH (0.06 nM) and pyruvate (10 mM) were incubated with 1 mM NADH in the presence of the indicated concentrations of PQQ at 37 °C for 2 h. The results shown are means ± SE (*n* = 3). (**d**) Effects of PQQ and PQQH_2_ on LDH activity in the reverse reaction (lactate + NAD^+^ → pyruvate + NADH). Rabbit muscle LDH (60 nM) and lactate (5 mM) were incubated with or without 0.25 mM NAD^+^ in the presence or absence of 50 μM PQQ or PQQH_2_ at 37 °C for 3 h, and then pyruvate production was determined by HPLC. The results shown are means ± SE (*n* = 3). *P < 0.05, **P < 0.01, ***P < 0.001 compared as indicated (ANOVA, Tukey-Kramer test). (**e**) PQQ-dependent promotion of reverse reaction of LDH. Rabbit muscle LDH (60 nM) and lactate (5 mM) were incubated with 0.25 mM NAD^+^ in the presence of the indicated concentrations of PQQ at 37 °C for 5 h. The results shown are means ± SE (*n* = 3).

LDH requires NADH/NAD^+^ cofactor to catalyze bidirectional conversion of pyruvate and l-lactate (Fig. 3a). In the forward reaction, pyruvate is reduced to produce l-lactate while reduced NADH is oxidized to NAD^+^ (pyruvate + NADH → lactate + NAD^+^). This reaction is overwhelmingly favored. In the reverse reaction, l-lactate is oxidized to form pyruvate whereas NAD^+^ is reduced to NADH (lactate + NAD^+^ → pyruvate + NADH). Hence, we examined the effects of PQQ/PQQH_2_ on LDH activity in the presence or absence of NADH/NAD^+^ cofactor. We incubated rabbit muscle LDH with pyruvate in the presence and absence of PQQ or PQQH_2_ in sodium phosphate buffer (pH 7.4) at 37 °C and measured lactate. As shown in Fig. 3b, neither PQQ nor PQQH_2_ served as an alternative cofactor for the reduction of pyruvate by LDH. However, both PQQ and PQQH_2_ inhibited the formation of lactate from pyruvate in the presence of NADH (i.e., forward reaction of LDH). In addition, the inhibition was dependent on PQQ concentration (Fig. 3c). On the other hand, PQQ and PQQH_2_ greatly enhanced the production of pyruvate from l-lactate in the presence of NAD^+^ (i.e., reverse reaction of LDH), although pyruvate production was not observed in the absence of NAD^+^ (Fig. 3d). We also observed that PQQ enhanced pyruvate formation by LDH in the presence of NAD^+^ (Fig. 3e).

### Characterization of PQQ-dependent LDH reaction

**Figure 4:**
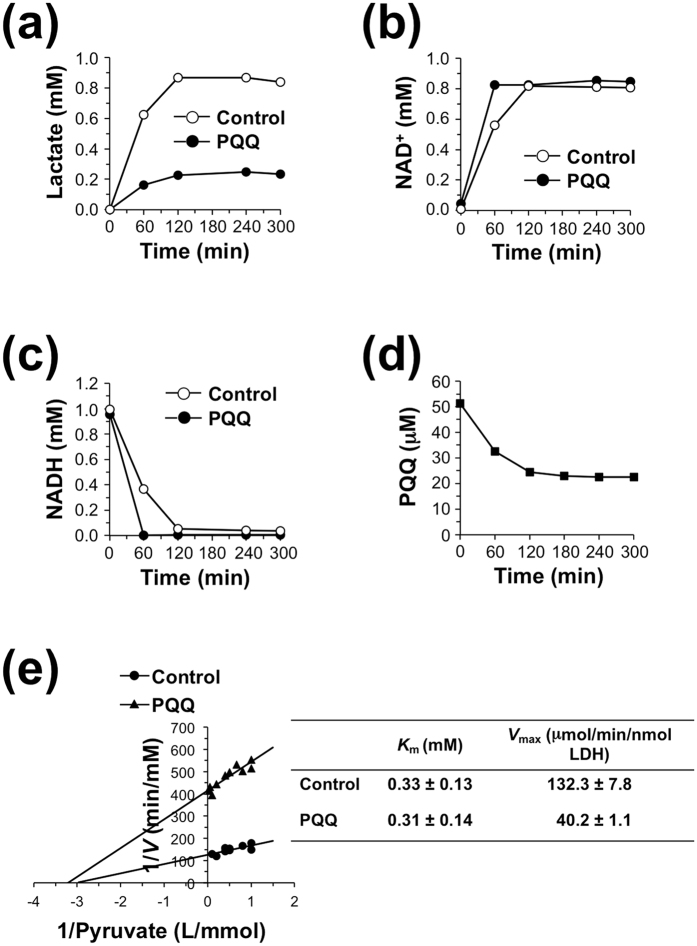
Time course of the forward reaction of LDH with PQQ. Rabbit muscle LDH (0.06 nM) and pyruvate (10 mM) were incubated with 1 mM NADH in the presence or absence of 50 μM PQQ at 37 °C for the indicated time. Then, concentrations of lactate (**a**), NAD^+^ (**b**), NADH (**c**), and PQQ (**d**) in the reaction mixtures were determined by HPLC. (**e**) Lineweaver-Burk plot of LDH reaction for pyruvate in the presence or absence of 50 μM PQQ.

To characterize the mechanism underlying the PQQ-mediated regulation of LDH activity, we determined the product and cofactor during the enzymatic reaction of LDH. The forward reaction of rabbit muscle LDH was performed in sodium phosphate buffer (pH 7.4), containing 10 mM pyruvate and 1 mM NADH in the presence or absence of 50 μM PQQ at 37 °C. The addition of PQQ to the reaction mixture suppressed lactate formation (Fig. 4a), whereas NAD^+^ formation and NADH oxidation were accelerated by PQQ (Fig. 4b,c). Of note, lactate production and NADH oxidation in the presence of PQQ were not stoichiometrically linked. There was only a modest decrease in PQQ accompanying the generation of lactate and NAD^+^, compared with the complete loss of NADH (Fig. 4d). We also determined the kinetic parameters for PQQ-mediated reaction of LDH from the Lineweaver–Burk plots and observed that, although the forward reaction in the presence of PQQ showed much lower *V*_max_ than the control reaction, the *K*_m_ values against pyruvate were almost unchanged in the presence and absence of PQQ (Fig. 4e).

**Figure 5:**
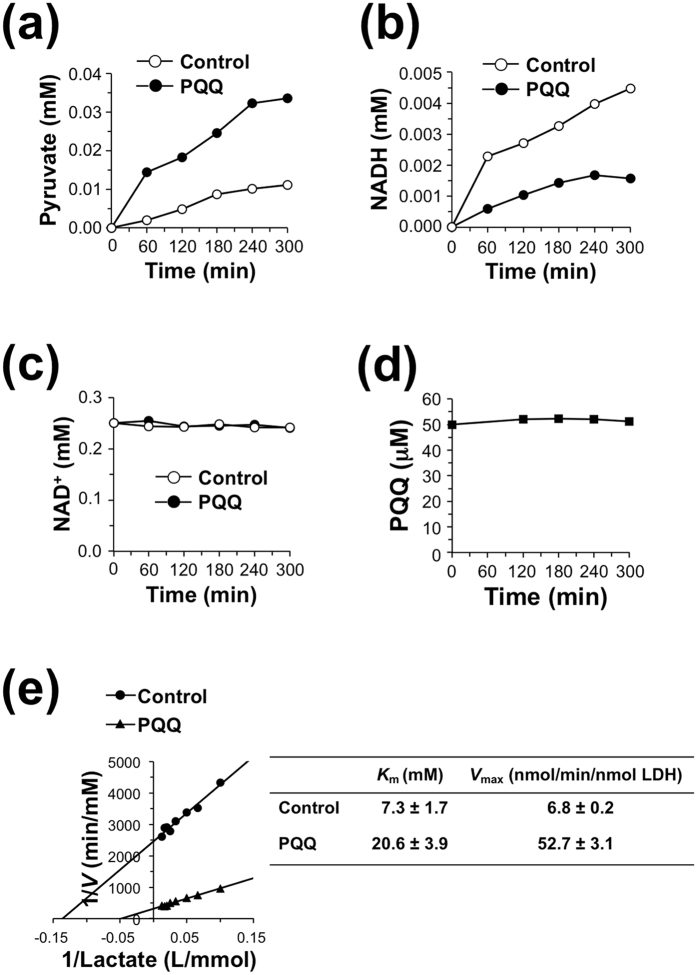
Time course of the reverse reaction of LDH with PQQ. Rabbit muscle LDH (60 nM) and lactate (5 mM) were incubated with 0.25 mM NAD^+^ in the presence or absence of 50 μM PQQ at 37 °C for the indicated time. Then, concentrations of pyruvate (**a**), NADH (**b**), NAD^+^ (**c**), and PQQ (**d**) in the reaction mixtures were determined by HPLC. (**e**) Lineweaver-Burk plot of LDH reaction for lactate in the presence or absence of 50 μM PQQ.

Then, we performed a comprehensive analysis of the effect of PQQ on the reverse reaction of LDH using l-lactate as a substrate and NAD^+^ as a cofactor (Fig. 5). Rabbit muscle LDH was incubated with 5 mM l-lactate and 0.25 mM NADH in the presence or absence of 50 μM PQQ. As shown in Fig. 5a, PQQ greatly facilitated the production of pyruvate. The formation of NADH was significantly suppressed in the presence of PQQ (Fig. 5b,c), while the concentration of PQQ was not altered during the reaction (Fig. 5d). The reverse reaction in the presence of PQQ showed much higher *V*_max_ compared to the control reaction, but the *K*_m_ value against lactate was markedly increased by the addition of PQQ (Fig. 5e). These data suggest that PQQ might enhance pyruvate production and suppress lactate production by modulating LDH activity.

On the other hand, the PQQ-dependent alteration in *V*_max_ of both forward and reverse reactions of LDH were not correlated with *K*_m_. In addition, the LDH reaction in the presence of PQQ resulted in a non-stoichiometric decline in NADH cofactor (data not shown). These data suggest that PQQ might oxidize NADH to generate NAD^+^ during the enzymatic reaction and promote the NAD^+^-dependent oxidation of l-lactate to form pyruvate.

### Oxidation of NADH by PQQ

**Figure 6:**
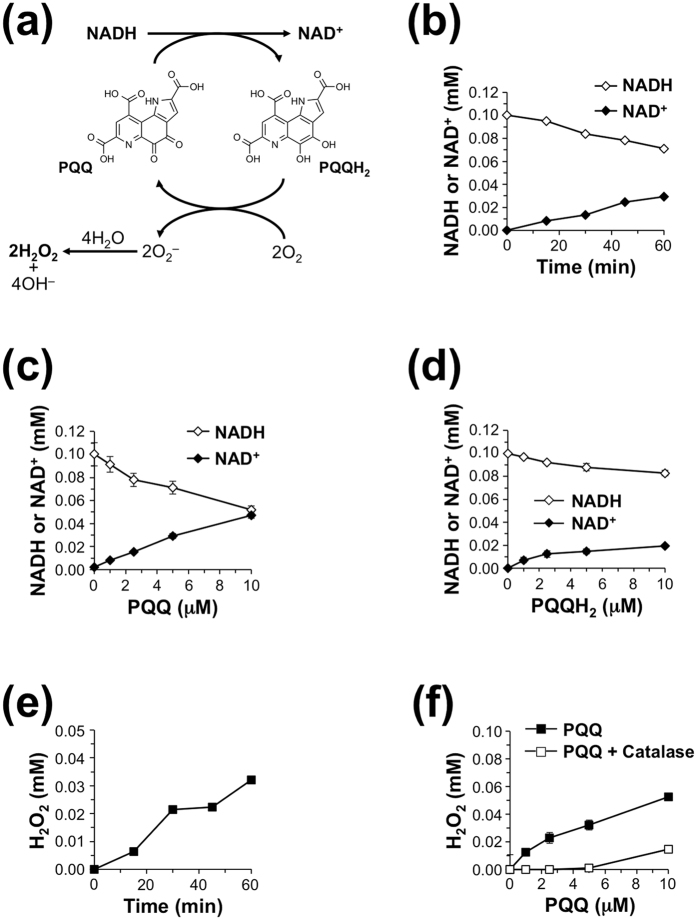
Formation of NAD^+^ by the PQQ-catalyzed oxidation of NADH. (**a**) Scheme for the mechanism underlying PQQ-catalyzed oxidation of NADH *via* redox cycling. (**b**) Time course of NAD^+^ formation by the reaction of PQQ with NADH. PQQ (5 μM) was incubated with 0.1 mM NADH in 0.1 M sodium phosphate buffer (pH 7.4) at 37 °C for the indicated time. (**c**) PQQ-dependent formation of NAD^+^ by the reaction with NADH. The indicated concentrations of PQQ were incubated with 0.1 mM NADH in 0.1 M sodium phosphate buffer (pH 7.4) at 37 °C for 60 min. The results shown are means ± SE (*n* = 3). (**d**) PQQH_2_-dependent formation of NAD^+^ by the reaction with NADH. The indicated concentrations of PQQH_2_ were incubated with 0.1 mM NADH in 0.1 M sodium phosphate buffer (pH 7.4) at 37 °C for 60 min. The results shown are means ± SE (*n* = 3). (**e**) Time course of H_2_O_2_ formation by incubation of NADH with PQQ. PQQ (5 μM) was incubated with 0.1 mM NADH in 0.1 M sodium phosphate buffer (pH 7.4) at 37 °C for the indicated time. The concentration of H_2_O_2_ was measured by the FOX assay. (**f**) PQQ-dependent formation of H_2_O_2_ by incubation with NADH. The indicated concentrations of PQQ were incubated with 0.1 mM NADH in 0.1 M sodium phosphate buffer (pH 7.4) in the presence or absence of 40 U/mL catalase at 37 °C for 60 min. The results shown are means ± SE (*n* = 3).

PQQ is known to mediate an electron transfer from a number of organic substrates^3^. As illustrated in Fig. 6a, a redox reaction between PQQ and NADH occurs to give PQQH_2_ and NAD^+^, respectively^21^. To evaluate the redox potency of PQQ to yield NAD^+^ from NADH, we incubated 5 μM PQQ with 0.1 mM NADH in sodium phosphate buffer (pH 7.4) at 37 °C and determined NAD^+^ formation. As shown in Fig. 6b,c, PQQ oxidized NADH to generate the corresponding NAD^+^ in a time- and concentration-dependent manner. In addition, the yield of NAD^+^ was greater than the amount of the added PQQ after 60 min of incubation. These data suggest that PQQ catalyzes the oxidation of NADH through its redox cycling reaction.

On the other hand, PQQH_2_ generated in the process of redox cycling is readily oxidized back to the original quinone *via* the reduction of two equivalents of molecular oxygen (O_2_) to superoxide anion (O_2_^−^), which spontaneously dismutates to hydrogen peroxide (H_2_O_2_) and OH^−^ (Fig. 6a)^21^,^22^. As shown in Fig. 6d, we also confirmed that the incubation of NADH with PQQH_2_ elicited concentration-dependent formation of NAD^+^ with a concomitant decrease in NADH. Furthermore, we observed time- and concentration-dependent accumulation of H_2_O_2_ in the incubation of NADH with PQQ (Fig. 6e,f). These data indicate that PQQ catalyzes the oxidation of NADH by its continuous redox cycling.

### Regulation of LDH activity by PQQ

**Figure 7:**
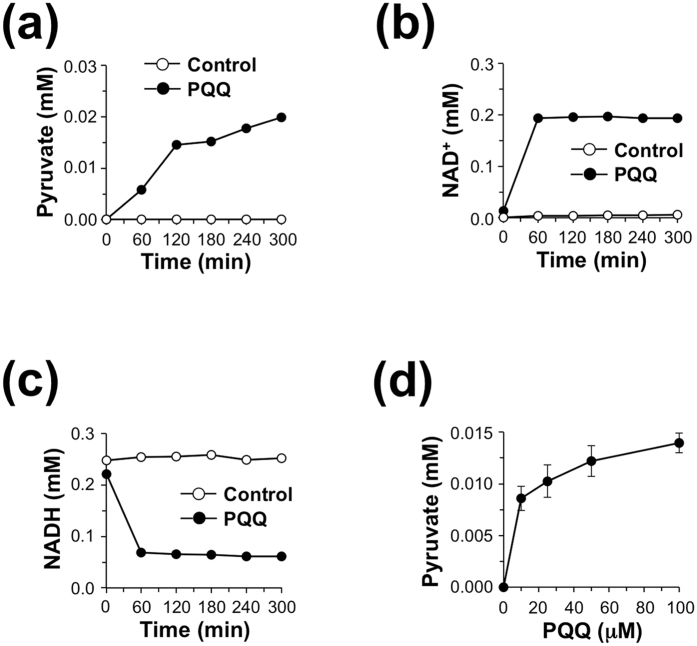
Time course of pyruvate formation by LDH in the presence of NADH and PQQ. (**a–d**) Rabbit muscle LDH (60 nM) and lactate (5 mM) were incubated with 0.25 mM NADH in the presence or absence of 50 μM PQQ at 37 °C for the indicated time. Then, concentrations of pyruvate (**a**), NAD^+^ (**b**), and NADH (**c**) in the reaction mixtures were determined by HPLC. (**d**) PQQ-dependent formation of pyruvate by LDH in the presence of NADH. Rabbit muscle LDH (60 nM) and lactate (5 mM) were incubated with 0.25 mM NADH in the presence of the indicated concentrations of PQQ at 37 °C for 5 h. The results shown are means ± SE (*n* = 3).

The results obtained so far suggest that the promotion of pyruvate formation and suppression of lactate formation by PQQ/LDH may be mediated via the redox-cycling activity of PQQ. To prove this hypothesis, we incubated rabbit muscle LDH with l-lactate and NADH in the presence or absence of PQQ and conducted a kinetic analysis. As shown in Fig. 7a, LDH did not catalyze the production of pyruvate in the absence of PQQ whereas, in the presence of PQQ, a significant amount of pyruvate was generated in a time-dependent manner. Consistently, we also observed the oxidation of NADH to produce NAD^+^ in the presence of PQQ (Fig. 7b,c). The formation of pyruvate was also dependent on the concentration of PQQ (Fig. 7d). These data support our hypothesis that the PQQ-mediated regulation of LDH activity could be attributed to the oxidation of NADH to NAD^+^ *via* the redox-cycling activity of PQQ.

**Figure 8:**
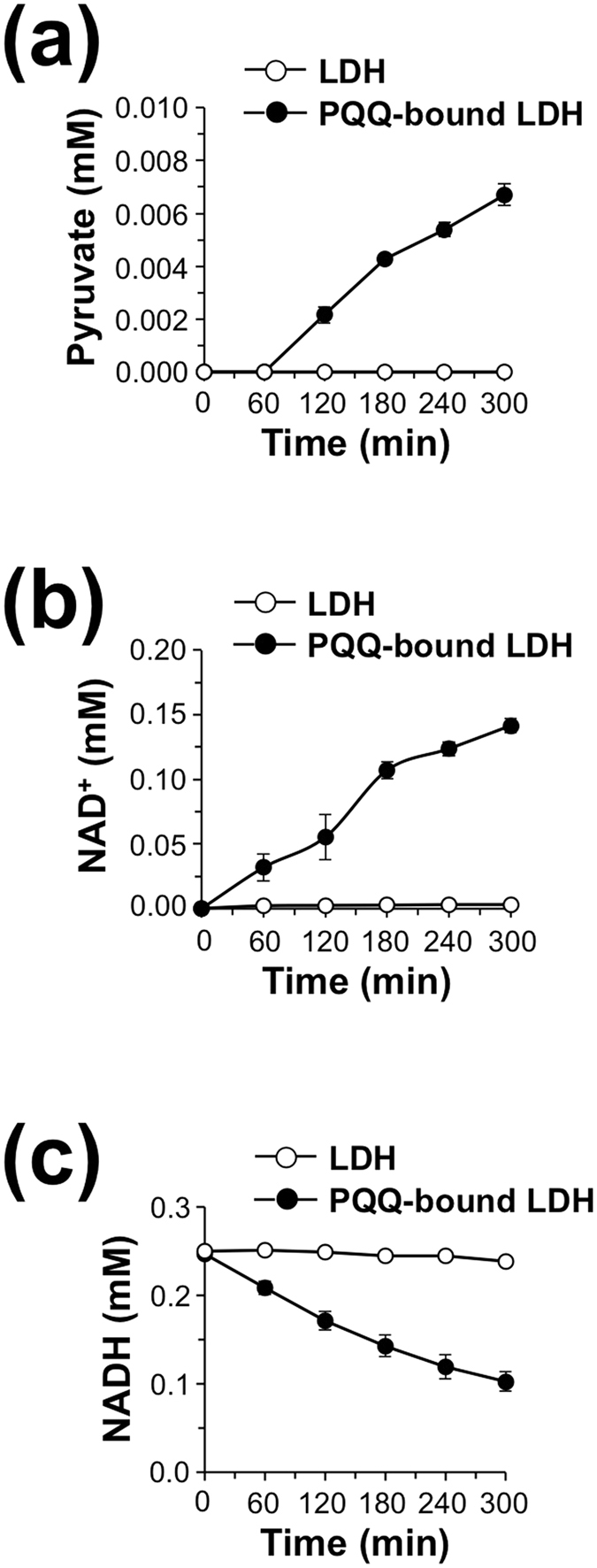
Time course of pyruvate formation by PQQ-bound LDH in the presence of NADH. Rabbit muscle LDH (600 nM) and PQQ-bound LDH (600 nM) were incubated with 0.25 mM NADH and 5 mM lactate at 37 °C for the indicated time. Then, concentrations of pyruvate (**a**), NAD^+^ (**b**), and NADH (**c**) in the reaction mixtures were determined by HPLC. The results shown are means ± SE (*n* = 3).

We next studied the conversion of l-lactate to pyruvate by the LDH-bound form of PQQ. To evaluate whether the PQQ-bound LDH could potentiate the enzymatic activity of lactate conversion into pyruvate *via* its redox-cycling activity, we determined pyruvate concentration upon incubation of PQQ-bound LDH with l-lactate and NADH. We prepared PQQ-bound LDH by incubation of rabbit muscle LDH with PQQ, followed by dialysis to remove free PQQ, and confirmed that the PQQ-bound LDH alone oxidized NADH to NAD^+^ in a time-dependent manner (Fig. S2). As shown in Fig. 8a, the PQQ-bound LDH, but not intact LDH, significantly catalyzed the conversion of l-lactate to pyruvate in the presence of NADH. Concurrently, we observed the formation of NAD^+^ with decreasing NADH in the incubation of PQQ-bound LDH (Fig. 8b,c).

**Figure 9:**
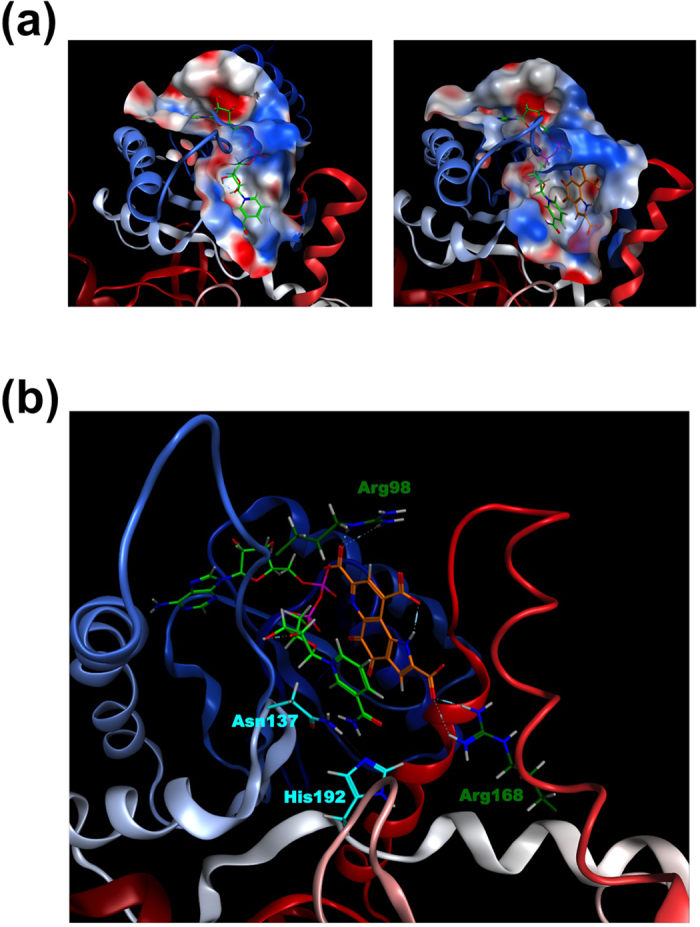
Docking simulations of PQQ to LDH-A. NADH (carbon atoms in *light green*) and PQQ (carbon atoms in *orange*) are shown in stick and color-coded by atom type (oxygen in *red*; nitrogen in *blue*). (**a**) *Left*, close up view of the active site in LDH-A with NADH. *Right*, The energy-minimized model of the ternary complex of LDH-A, NADH, and docked PQQ. The electrostatic potential is represented on a color scale from blue for a positive potential, white for neutral, to red for a negative potential. (**b**) The electrostatic interaction between LDH-A and docked PQQ. Electrostatic interactions between Arg-98 (carbon atoms in *green*) and 7-COOH group of PQQ and between Arg-168 (carbon atoms in *green*) and 2-COOH group of PQQ are shown as dashed lines.

To gain structural insight into the PQQ-bound LDH, we performed molecular docking simulation of PQQ into the apo structure of human LDH-A using MOE software. PQQ was docked at a position in the NADH-binding pocket of LDH-A where there was small overlap between docked PQQ and protein-bound NADH, the binary-complex structure of which is provided by the ligand soaking experiment (Fig. 9a)^23^. The energy-minimized structure of the ternary complex comprised of LDH-A, NADH, and docked PQQ (Fig. 9a) indicated that the substrate pocket is large enough to fit NADH and PQQ simultaneously without significant conformational changes of the enzyme. The quinone moiety of PQQ was situated in close proximity to the reduced nicotinamide moiety of NADH in a plane-parallel manner. Two pairs of electrostatic interactions between Arg-98 and the 7-COOH group of PQQ and between Arg-168 and the 2-COOH group of PQQ are predicted, which seem to play a role in PQQ binding in the pocket (Fig. 9b). As seen in Fig. S1, the putative amino acid residues (Arg-98 and Arg-168) involved in PQQ binding are also completely conserved in mouse and rabbit LDH-A. On the other hand, the substrate-binding cavity and putative substrate-binding residues at positions 137 and 192^24^ were well-kept. These data suggest that the docked PQQ molecule might cause the oxidation of NADH cofactor at the active site of LDH-A and promote the NAD^+^-dependent oxidation of l-lactate.

**Figure 10:**
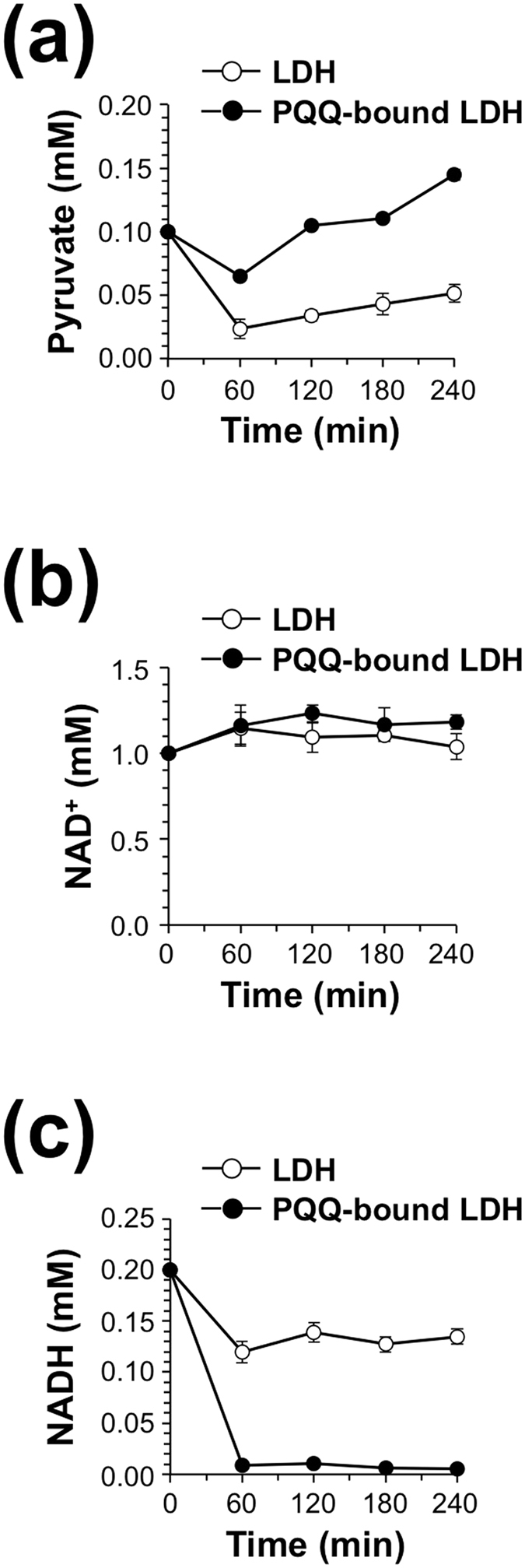
Effect of PQQ binding on chemical equilibrium of LDH reaction. Rabbit muscle LDH (600 nM) and PQQ-bound LDH (600 nM) were incubated with 10 mM lactate and 0.1 mM pyruvate in the presence of 0.2 mM NADH and 1 mM NAD^+^ at 37 °C for the indicated time. Then, concentrations of pyruvate (**a**), NAD^+^ (**b**), and NADH (**c**) in the reaction mixtures were determined by HPLC. The results shown are means ± SE (*n* = 4).

Under physiological conditions, the ratio of cytosolic free NAD^+^/NADH varies from 1 to 700^25^, while intracellular lactate/pyruvate ratio has been reported to be about 50–200^26^. Normally, the cellular pyruvate and NADH concentrations are lower than lactate and NAD^+^ concentrations. The cellular lactate/pyruvate ratio reflects the redox potential of the cell and delineates the balance between NAD^+^ and NADH, which is highly dependent on the interconversion of lactate and pyruvate *via* LDH activity^25^,^26^,^27^. Hence, we tested the effect of PQQ binding to LDH on chemical equilibrium of the LDH reaction. As shown in Fig. 10a, the PQQ-bound LDH maintained higher levels of pyruvate than the intact LDH throughout the incubation time. However, the PQQ-bound LDH drastically decreased the level of NADH as compared with the intact LDH during the incubation period (Fig. 10b,c), suggesting that the PQQ-bound LDH oxidizes NADH to form NAD^+^ more efficiently than LDH and thereby shifts the equilibrium of LDH toward pyruvate production by oxidation of lactate. These data suggest that PQQ could cause the acceleration of l-lactate oxidation to pyruvate by targeting LDH-A in cells.

### Suppression of cellular lactate production by PQQ

**Figure 11:**
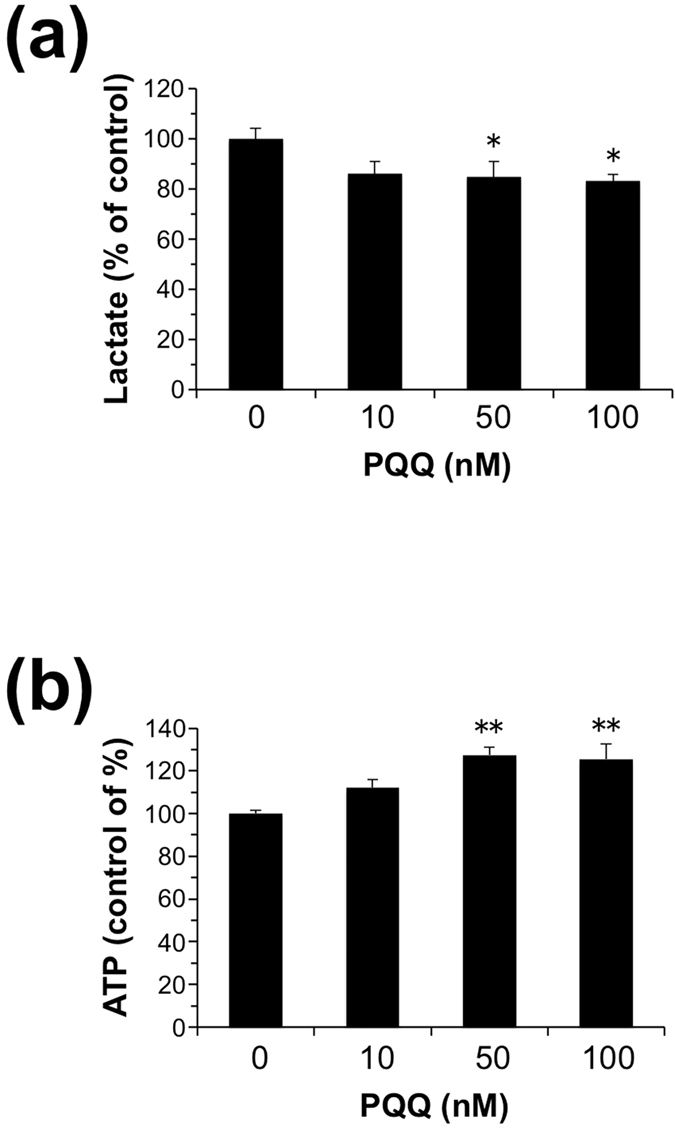
Effect of PQQ on production of lactate and ATP in NIH/3T3 fibroblasts. The cells were incubated with the indicated concentrations of PQQ in serum- and pyruvate-free DMEM for 24 h. Then, lactate levels in the culture media (**a**) and cellular ATP levels (**b**) were determined as described in the Experimental Procedures. The results shown are means ± SE (*n* = 6). *P < 0.05, **P < 0.01 *versus* vehicle-treated control (ANOVA, Dunnett’s multiple comparison test).

Pyruvate generated in glycolysis is preferentially converted to l-lactate by LDH-A, and the excess lactate is released into the extracellular space *via* monocarboxylate transporters^28^. Meanwhile, pyruvate is also metabolized *via* the tricarboxylic acid (TCA) cycle. To evaluate the physiological significance of PQQ-dependent LDH reaction, we examined lactate release into the cell culture medium in the NIH/3T3 fibroblasts exposed to PQQ. As shown in Fig. 11a, the treatment of the cells with 50 nM PQQ and above in serum- and pyruvate-free DMEM for 24 h significantly decreased lactate formation in the culture media by about 85% of the control, suggesting that PQQ might inhibit the forward reaction and/or promote the reverse reaction by LDH. Because pyruvate is oxidatively decarboxylated by the pyruvate dehydrogenase complex to form acetyl-CoA followed by ATP production in the TCA cycle, we speculated that the PQQ treatment might give rise to enhanced production of cellular ATP. Indeed, the exposure of NIH/3T3 cells to PQQ for 24 h caused elevation of intracellular ATP levels in a dose-dependent manner (Fig. 11b). Thus, PQQ might modulate the lactate and pyruvate metabolism by its redox activity, leading to enhanced energy production *via* oxidative phosphorylation in the TCA cycle.

## Discussion

It is widely recognized that PQQ is an important nutrient for growth and development in animals^1^,^2^,^8^,^9^. However, detailed mechanisms for the vitamin-like activity of PQQ remain unclear. In the present study, to gain an insight into the molecular mechanism underlying its physiological and nutritional functions, we sought to identify PQQ target proteins using a proteomics approach based on an affinity pull-down with PQQ-Sepharose beads and successfully identified three key enzymes (pyruvate kinase, LDH, and triosephosphate isomerase) involved in glycolysis, one antioxidant enzyme (peroxiredoxin), one key enzyme in nucleotide metabolism (nucleoside diphosphate kinase), and one translation elongation factor as the PQQ-binding proteins in the cell lysates from NIH/3T3 cells (Fig. 2a, Tables 1 and 2). Among these proteins, we focused on LDH and indeed showed that, despite the absence of known PQQ-binding motifs, the purified LDH (from rabbit muscle) bound PQQ (Fig. 2b,c).

**Figure 12:**
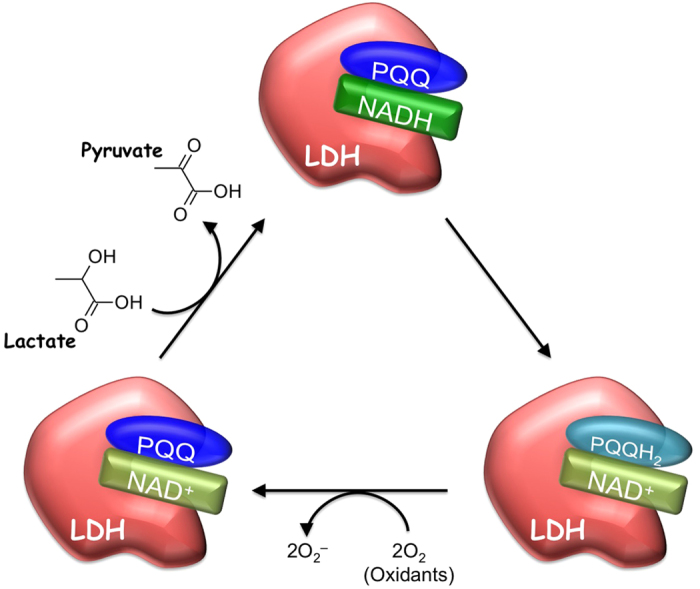
Proposed mechanism for PQQ-dependent enzymatic reaction of LDH *via* its redox activity.

LDH (EC 1.1.1.27) is a homo- or hetero-tetrameric enzyme composed of two subunits, LDH-A (so-called muscle type) and LDH-B (so-called heart type), encoded by two highly related genes^29^. This study proved for the first time that PQQ enhances the enzymatic activity of mammalian LDH, converting l-lactate to pyruvate through the oxidation of NADH to NAD^+^ (Fig. 12). It is noteworthy that PQQ in the LDH-bound form maintains its redox properties (Fig. S2), contributing to the oxidation of lactate in the presence of NADH (Fig. 8). Moreover, molecular docking studies showed that PQQ could be positioned near the NADH cofactor in the active site pocket of LDH-A (Fig. 9). These results suggest that PQQ might interact with NADH in the active site of LDH-A, leading to the formation of NAD^+^. The oxidation of lactate to pyruvate is accompanied by the reduction of NAD^+^ to NADH and the generated pyruvate can be favorably reverted to lactate by LDH. However, the PQQ bound to LDH can reoxidize NADH to NAD^+^ with concomitant formation of the hydroquinone derivative PQQH_2_, leading to inhibition of the reverse reaction and further oxidation of lactate by LDH. Subsequently, aerobic auto-oxidation of PQQH_2_ yields O_2_^−^ and regenerates PQQ as the NADH-oxidation catalyst. PQQH_2_ can also be reoxidized to PQQ *via* the reaction with radical species such as singlet oxygen, aroxyl radical, and peroxyl radical, and acts as a potent radical scavenger^30^,^31^,^32^,^33^. PQQ has a much higher redox potential (+0.090 V; *vs*. SHE) than NAD^+^ (−0.320 V; *vs*. SHE) and is capable of carrying out thousands of redox catalytic cycles at neutral pH and moderate temperatures^1^,^34^,^35^,^36^. Even though other quinone biofactors are liable to either self-oxidize or condense into an inactive form, PQQ is relatively stable and does not easily polymerize during redox cycling. Therefore, PQQ can stably catalyze the oxidation of NADH through its continuous and repeated redox cycling, and thereby effectively enhance the enzymatic activity of LDH-mediated lactate-to-pyruvate conversion. Although the detailed mechanism is not fully elucidated, the redox property of PQQ bound to LDH might, at least in part, be involved in the enhanced activity of LDH to convert lactate to pyruvate. Future studies are need to define the contribution of the binding of PQQ to LDH in this newly established PQQ-dependent enzymatic reaction.

It is also noted that the treatment of NIH/3T3 fibroblasts with 50 nM PQQ significantly reduced cellular lactate release (Fig. 11a). The mean maximum level of free PQQ in human and rat tissues was reported to be about 30 nM^5^,^37^. Therefore, the concentrations of PQQ used in this study are physiologically relevant. Moreover, this observation implies that PQQ might facilitate the conversion of lactate to pyruvate through binding to cellular LDH. On the other hand, cytosolic free PQQ might facilitate the oxidation of NADH to NAD^+^ *via* its redox activity. In the present study, we also observed that the forward reaction of rabbit muscle LDH was significantly inhibited by the presence of PQQ (Fig. 4). Hence, PQQ might suppress the LDH-catalyzed conversion of pyruvate to lactate by decreasing NADH concentration, or by inhibiting the binding of NADH in the cells. Increased pyruvate levels are anticipated to shift the overall equilibrium toward acetyl-CoA formation from pyruvate, leading to enhanced ATP generation by oxidative phosphorylation in the mitochondrial TCA cycle. Furthermore, in the glycolytic pathway, one glucose molecule is catabolized to two pyruvate molecules using 2 ATP and 2 NAD^+^ while producing 4 ATP and 2 NADH molecules. LDH-A regulates the last step of glycolysis that preferentially generates lactate and permits the regeneration of NAD^+^. Therefore, cytosolic free PQQ might also enhance the generation of ATP and pyruvate in glycolytic pathway by increasing NAD^+^ levels. Indeed, we showed that the exposure of NIH/3T3 cells to PQQ results in a significant increase in intracellular ATP levels (Fig. 11b). Glycolysis and oxidative phosphorylation are two major metabolic pathways for producing ATP in mammalian cells. Energy consumption from metabolic activities in normal cells relies primarily on mitochondrial oxidative phosphorylation, which is efficient and generates more ATP than glycolysis. Because mitochondria function as the principal energy source of the cell, compromised function of this key organelle is linked to numerous diseases and metabolic disorders.

Recent studies have shown that lowering the activity of mitochondrial oxidative phosphorylation and ATP production causes cellular senescence^38^, neurodegenerative diseases^39^, and diabetes mellitus^40^. In addition, persistent reduction of mitochondrial oxidative phosphorylation activity is associated with the release of oxidants from nonmitochondrial sources, release of proinflammatory and profibrotic cytokines, and manifestation of organ dysfunction^41^. PQQ deficiency in mice reduces hepatic mitochondrial content by 20–30%, and suppresses mitochondrial respiration^42^, whereas PQQ administration reverses the mitochondrial changes and metabolic disorders, and significantly improves lipid profiles in a rat model of type 2 diabetes^37^,^42^. Moreover, dietary PQQ supplementation has been shown to enhance mitochondrial function and biogenesis and improve metabolic homeostasis in mice and rats^37^,^42^,^43^. It is also noteworthy that increasing concentrations of pyruvate enhance mitochondrial biogenesis, basal respiratory rate, and maximal oxidative capacity in cultured myoblasts^44^,^45^. These findings and our observations together suggest that the PQQ-dependent modulation of LDH activity to facilitate the formation of pyruvate might be involved in the PQQ-inducible mitochondrial biogenesis and increased mitochondrial respiration. PQQ may therefore have therapeutic potential for various acute and chronic diseases including neurodegenerative, metabolic, and mitochondrial diseases.

In conclusion, we identified LDH-A as one of the major PQQ-binding proteins using PQQ-immobilized affinity beads and demonstrated the direct binding of PQQ to rabbit muscle LDH. Of note, PQQ augments the enzymatic activity of LDH to convert l-lactate to pyruvate *via* the oxidation of NADH to NAD^+^ by its redox-cycling activity. Although the exact location of the binding site remains unclear, the PQQ binding to LDH also facilitated the enzymatic oxidation of lactate to pyruvate *via* its redox activity. Moreover, our results showed that the exposure of NIH/3T3 fibroblasts to PQQ significantly attenuates cellular lactate release and coincidently increases intracellular ATP level. This finding suggests that PQQ may potentiate the enzymatic activity of cellular LDH of converting lactate into pyruvate, at least in part, through binding to LDH, leading to enhanced ATP production *via* the mitochondrial TCA cycle and oxidative phosphorylation. Future studies are need to evaluate the contribution of this newly discovered PQQ-dependent enzymatic reaction to the important nutritional and physiological functions of PQQ, including cellular energy metabolism and mitochondrial biogenesis in mammals.

## Methods

### Chemicals

PQQ disodium salt and PQQH_2_ were provided by Mitsubishi Gas Chemical Company Inc. (Tokyo, Japan). EAH-Sepharose 4B (ω-aminohexyl-Sepharose 4B) was obtained from GE Healthcare UK Ltd. (Buckinghamshire, UK). DEAE-Cellulofine AM was obtained from Seikagaku Kogyo (Tokyo, Japan). Keyhole limpet hemocyanin (KLH), 1-ethyl-3-[3-dimethylaminopropyl]carbodiimide hydrochloride (EDC), and *N*-hydroxysuccinimide (NHS) were purchased from Thermo Fisher Scientific (Waltham, MA, USA). Charcoal stripped fetal bovine serum (FBS-C) was obtained from Life Technologies (Carlsbad, CA, USA). Phenazine methosulfate (PMS), nitrotetrazolium blue (NTB), dithiothreitol (DTT), protease inhibitor cocktail, tris-(2-carboxyethyl)phosphine hydrochloride (TCEP), trifluoroacetic acid (TFA), 2-(*N*-morpholino)ethanesulfonic acid (MES), iodoacetamide, Dulbecco’s modified Eagle’s medium (DMEM), penicillin, streptomycin, rabbit muscle LDH, lithium l-lactate, sodium pyruvate, NADH, and NAD^+^ were purchased from Nacalai Tesque (Kyoto, Japan). A PQQ-dependent enzyme, glucose dehydrogenase (GDH), from *Acinetobacter calcoaceticus* was obtained from Toyobo (Osaka, Japan).

### Preparation of PQQ-immobilized beads (PQQ-Sepharose)

The scheme of immobilization is shown in Fig. 1a. EAH-Sepharose 4B (ω-aminohexyl-Sepharose 4B) beads were washed five times with ~10 bed volumes of 100 mM MES buffer containing 0.5 M NaCl (pH 4.5). Then, the beads (approx. 200 μL) were mixed with 250 μL of 8 mM PQQ, 75 μL of 266 mM NHS, 300 μL of 133 mM EDC. The mixture was incubated for 15 min at room temperature with shaking and then overnight at 4 °C with rotary shaking. The resulting beads were washed five times with ~10 bed volumes of 100 mM MES buffer containing 0.5 M NaCl (pH 4.5) and stored at 4 °C.

### Preparation of the apo form of PQQ-dependent GDH

apo-GDH was prepared according to the published procedure^46^,^47^ with some modification. A 45 mL volume of 50 mM PIPES-NaOH buffer (pH 6.5) containing DEAE-Cellulofine AM gel (2.5 mL of settled gel) was heated to 50 °C. Then, 1.1 mg of holo-GDH in 90 μL of 50 mM PIPES-NaOH buffer (pH 6.5) was added. The suspension was slowly agitated and incubated for 20 min at 50 °C. After further incubation at room temperature for 20 min the anion-exchange resin was removed by filtration (0.2 μm pore size). To concentrate the protein, the supernatant was concentrated to 1.5 mL by ultrafiltration in a Viva spin 20 (10,000 MWCO) concentrator (Sartorius, Göttingen, Germany). The preparation was dialyzed for 14 h against 50 mM PIPES-NaOH buffer (pH 6.5), diluted with the same buffer containing 1 mM CaCl_2_, 0.1% Triton X-100, and 0.1% bovine serum albumin (BSA). The resulting apo-GDH was stored at 4 °C. We confirmed that apo-GDH was almost completely inactivated by measuring GDH activity.

### Enzyme assay for GDH

GDH activity was determined by measuring diformazan produced following the reduction of NTB with PMS in the presence of d-glucose^48^. Before enzymatic reactions, 1 mL of 50 mM PIPES-NaOH buffer (pH 6.9) containing 0.22% Triton X-100, 35 μL of 1 M d-glucose solution, 58 μL of 3 mM PMS solution, and 39 μL of 6.6 mM NTB solution were mixed and preheated at 37 °C for 5 min, and then, 180 μL of the mixed substrate solution was added to each well of a 96-well microplate with 10 μL of an enzyme solution. After gentle mixing, diformazan produced by enzymatic reaction was measured at an absorbance of 570 nm using a 96-well microplate reader at intervals of 30 sec for 5 min.

### *In vitro* binding assay using EAH- and PQQ-Sepharose beads

EAH- and PQQ-Sepharose beads were equilibrated with 50 mM PIPES-NaOH buffer (pH 6.5). Proteins (0.1 mg) in 100 μL of 50 mM PIPES buffer (pH 6.5) containing 1 mM CaCl_2_ and 1 mM MgCl_2_ were incubated with EAH- and PQQ-Sepharose beads (20 μL) for 1 h at room temperature with rotary shaking. The beads were washed five times with 50 mM PIPES buffer (pH 6.5) containing 1 mM CaCl_2_ and 1 mM MgCl_2_, and bound proteins were eluted with the same buffer containing 1 mM PQQ. After mixing for 3 min and centrifugation at 16,000 × *g* for 10 min at 4 °C, the supernatants were analyzed by sodium dodecyl sulfate-polyacrylamide gel electrophoresis (SDS-PAGE).

### Preparation of anti-PQQ antibody

Rabbit anti-PQQ antibody was produced using PQQ-coupled KLH, and the antisera were produced by Medical and Biological Laboratories (Nagoya, Japan). The IgG fraction was isolated from the serum obtained using the Proteus Protein G Purification Kit (AbD Serotec, Kidlington, UK). PQQ-coupled KLH was prepared according to the published procedure^49^,^50^. Briefly, KLH (15 mg) was coupled with PQQ (1 mg) in the presence of NHS (60 mg) and EDC (30 mg) in 3 mL of phosphate-buffered saline (PBS) at room temperature in the dark for 5 h. Then, the mixture was dialyzed against PBS three times at 4 °C for 24 h. The specificity of the antibody’s affinity was assessed by enzyme-linked immunosorbent assay (ELISA).

### ELISA

A 100 μL aliquot of the sample solution containing 100 μg/mL rabbit muscle LDH and/or 1 mM PQQ was added to each well of a 96-well ELISA plate and incubated at 37 °C for 3 h. The solution was then removed, and the plate was washed with PBS containing 0.5% Tween 20 (PBS-T). Each well was incubated with 200 μL of 4% Blockace (Yukijirushi, Sapporo, Japan) in PBS-T for 60 min at 37 °C to block the unsaturated plastic surface. The plate was washed three times with PBS-T, and then 100 μL of anti-PQQ antibody (1:2,500 in PBS-T) was added to each well and incubated for 2 h at 37 °C. After discarding the supernatants and washing three times with PBS-T, 100 μL of a goat anti-rabbit IgG conjugated to horseradish peroxidase (Medical and Biological Laboratories) (1:5,000 in PBS-T) was added. After incubation for 1 h at 37 °C, the supernatant was discarded, and the plates were washed three times with PBS-T. The enzymatic reaction was performed using the ELISA POD Substrate TMB Kit (Nacalai Tesque), terminated by adding 50 μL of 2 M sulfuric acid, and the absorbance was measured at 450 nm.

### Cell culture

The mouse embryo fibroblast cell line NIH/3T3 was obtained from the Cell Resource Center for Biomedical Research, Tohoku University, Japan, and maintained in a 5% CO_2_ humidified atmosphere at 37 °C in DMEM supplemented with 10% FBS-C, 100 units/mL penicillin, and 100 μg/mL streptomycin. The cells were seeded into 15-cm Petri dishes and cultured until they reached 80% confluence. After serum starvation for 24 h, the cells were washed twice with ice-cold PBS and lysed with 50 mM PIPES buffer (pH 6.5) containing 1% Triton X-100, 1 mM CaCl_2_, 1 mM MgCl_2_, and 1 × protease inhibitor cocktail by sonication. The cell lysates were centrifuged at 16,000 × *g* for 10 min at 4 °C, and then the protein concentration was determined with BCA Protein Assay Reagent (Thermo Fisher Scientific).

### Affinity purification with PQQ-Sepharose beads

EAH- and PQQ-Sepharose beads were equilibrated with 50 mM PIPES-NaOH buffer (pH 6.5). Cell extracts (2 mg proteins) were incubated with EAH-Sepharose beads (300 μL) for 1 h at 4 °C with rotary shaking. After centrifugation at 16,000 × *g* for 10 min at 4 °C, one-half of the supernatant was incubated with EAH- and PQQ-Sepharose beads (80 μL) for 2 h at room temperature with rotary shaking. Each beads were washed four times with 50 mM PIPES buffer (pH 6.5) containing 1% Triton X-100, 1 mM CaCl_2_, and 1 mM MgCl_2_, and bound proteins were eluted with 80 μL of the same buffer containing 1 mM PQQ. After vortexing for 3 min and centrifugation at 16,000 × *g* for 10 min at 4 °C, the supernatants were analyzed by SDS-PAGE and nano-LC-ESI-Q-TOF-MS/MS.

### SDS-PAGE

Proteins were diluted with Laemmli’s reducing sample buffer and incubated at 98 °C for 3 min. Samples were subjected to SDS-PAGE analysis on 10% acrylamide gels. After electrophoresis, the gels were stained with CBB R-250 or EzStain silver staining kit (Atto, Osaka, Japan).

### Protein identification by nano-LC-ESI-Q-TOF-MS/MS analysis

Both EAH- and PQQ-Sepharose eluates were concentrated by precipitation using the 2-D Protein Clean-Up Kit (GE Healthcare), according to the manufacturer’s instructions. The pellet was dissolved in 40 μL of tris-HCl buffer (100 mM, pH 8.5) containing 8 M urea and 100 mM TCEP. After intermittent vortexing for 1 h at room temperature, 1 μL of 500 mM iodoacetamide was added and incubated for 1 h with shaking in the dark at room temperature, followed by the addition of 1 μL of 0.2 M DTT. The mixtures were then diluted with 120 μL of H_2_O to reduce the concentration of urea and proteolyzed with 100 ng of sequence grade modified trypsin (Promega, Madison, WI) in 50 mM NH_4_HCO_3_ buffer in the presence of 0.01% Protease MAX surfactant (Promega) overnight at 37 °C. The tryptic digests were acidified with 0.3 μL of TFA, and solid-phase extraction was performed using Monospin C18 (GL Science, Tokyo, Japan). The solid phase was conditioned with 200 μL of 100% acetonitrile, and then equilibrated with 200 μL of 0.1% TFA. The tryptic peptides were loaded onto the solid phase, and salts and urea from the reaction buffer were washed from the solid phase with 200 μL of 0.1% TFA. The peptides were eluted from the solid phase with 200 μL of 60% acetonitrile containing 0.1% TFA. Subsequently, the peptide samples were concentrated using a centrifugal evaporator. Mass spectrometry was performed using a Triple TOF^TM^ 5600 system (AB SCIEX, Concord, CAN), a hybrid triple quadrupole time-of-flight mass spectrometer equipped with an ESI source, and the mass range was set at *m/z* 100–1250. The conditions of the MS/MS detector were as follows: ion spray voltage, 2300 V; ion source gas, 20 psi; interface heater temperature 150 °C; curtain gas 20 psi. Nitrogen was used as the nebulizer and auxiliary gas. Two independent assays for MS analysis were conducted. Protein identification was performed using MASCOT with the Swiss-Prot database. Proteins with MASCOT scores ≥40 and with ≥3 peptide matches were considered to be positively identified. The proteins detected in both assays were used in the subsequent analysis.

### Enzymatic assay for LDH

LDH activity in the forward reaction was determined in 0.1 M sodium phosphate buffer (pH 7.4) containing 5 mM l-lactate and 0.25 mM NAD^+^ in the presence of various concentrations of PQQ. The reaction was initiated by the addition of rabbit muscle LDH (60 nM), and the mixture was incubated at 37 °C with shaking. LDH activity in the reverse reaction was determined at 37 °C in 0.1 M sodium phosphate buffer (pH 7.4) containing 10 mM pyruvate and 1 mM NADH in the presence of various concentrations of PQQ. The reaction was initiated by the addition of rabbit muscle LDH (0.06 nM), and the mixture was incubated at 37 °C with shaking. The concentrations of pyruvate, lactate, NAD^+^, and NADH were measured by HPLC as described below. To determine the Michaelis constant (*K*_*m*_) and maximum reaction rate (*V*_*max*_), the assay was performed with various concentrations of substrates toward LDH in 0.1 mM sodium phosphate buffer (pH 7.4) with or without 50 μM PQQ at 37 °C with shaking, and the experimental data were analyzed using Lineweaver–Burk plots.

### Determination of pyruvate, lactate, NAD^+^, and NADH

Pyruvate, lactate NAD^+^, and NADH were quantified by HPLC according to the published procedure^51^ with some modifications. An HPLC gradient pump (L-2310, Hitachi, Tokyo, Japan) was coupled with a Rheodyne 7125 injector equipped with a 20 μL sample loop (Rheodyne, Cotati, CA, USA), an L-4000 UV detector (Hitachi), and a 655A-52 column oven (Hitachi). The column oven temperature was set at 40 °C and detection was performed at 220 nm. The mobile phase consisted of a combination of Solvent A (0.1 M sodium phosphate buffer, pH 2.0) and Solvent B (HPLC-grade methanol). The HPLC was run at a flow rate of 0.8 mL/min with 100% A from 0 to 10 min, a linear gradient to 80% A from 10 to 20 min, and at 80% A from 20 to 25 min.

### Determination of PQQ

PQQ was measured by reversed-phase HPLC as follows. The mobile phase constituted of ultrapure water (Solvent A) and HPLC-grade methanol (Solvent B) at a constant solvent flow rate of 0.8 mL/min. The sample was injected into an HPLC apparatus with a C-18 reversed phase column (Cosmosil 5C_18_-AR-II, 4.6 × 250 mm, Nacalai Tesque) eluted with the following mobile gradient: 0–10 min, linear gradient from 90% Solvent A to 75% Solvent A. The chromatographic run was completed in 30 min, including rinsing of the column in 50% methanol and the re-equilibration step. The column oven temperature was set at 40 °C and detection was performed at 360 nm.

### Determination of H_2_O_2_

The concentration of H_2_O_2_ was measured by the ferrous ion oxidation-xylenol orange (FOX) assay^52^. Reagent A was 4.4 mM butylated hydroxytoluene (Nacalai Tesque) in HPLC-grade methanol; reagent B was 1.0 mM xylenol orange (Nacalai Tesque) and 2.56 mM ammonium ferrous sulfate in 250 mM H_2_SO_4_. One volume of reagent B was added to 9 volumes of reagent A to make the FOX reagent. The sample (500 μL) was added to the FOX reagent (3.0 mL). The mixture was vortexed for 5 sec and then incubated at room temperature for 30 min. Solutions were then centrifuged at 2,000 g for 10 min at room temperature, and the absorbance was measured at 560 nm. The FOX assay was calibrated using a standard H_2_O_2_ solution.

### Preparation of PQQ-bound LDH

LDH (2 μM) was incubated with 500 μM PQQ at 37 °C for 1 h in 0.1 M sodium phosphate buffer (pH 7.4). Then, the resulting protein was dialyzed for 24 h against three changes of PBS. The protein concentration was determined with BCA Protein Assay Reagent.

### Molecular docking

The crystal structure of human LDH-A apo form (Protein Data Bank identification number 4L4S) at 2.1 Å^23^ was obtained from the Research Collaboratory for Structural Bioinformatics database. Protein structures were viewed and manipulated using Molecular Operating Environment (MOE) software (Chemical Computing Group, Montreal, Quebec, Canada). For molecular dynamics simulation, hydrogen positions and ionization states were assigned using Protonate 3D application^53^. The protein structures were equilibrated using the Generalized Born solvation model^54^. Docking studies were performed using Site Finder and Dock applications in MOE software. The PQQ conformation database produced by the conformational search (2 entries) was used to analyze for the query of the docking experiment. The ranking of the generated solutions was performed using the estimated free binding energy ΔG of the protein-ligand complex.

### Determination of lactate and ATP levels

NIH/3T3 fibroblasts were seeded into a 96-well culture plate and cultured until they reached 70–80% confluence. The cells were washed with serum- and pyruvate-free DMEM and then starved in the medium. Twenty-four hours later, the medium was exchanged with fresh serum- and pyruvate-free medium, and then the cells were exposed to PQQ. After 24 h of incubation, lactate levels in the culture media were determined using a lactate assay kit (Kyowa Medex, Tokyo, Japan) as described by the manufacturer’s instructions. Cellular ATP levels were determined using an ATP luminescence kit (TOYO B-Net, Tokyo, Japan) according to the manufacturer’s instructions. Lactate and cellular ATP levels were normalized to cell number. Cell number was determined by WST-8 reduction assay using a Cell Count Reagent SF kit (Nacalai Tesque) according to the manufacturer’s instructions.

## Additional Information

**How to cite this article**: Akagawa, M. *et al*. Identification of lactate dehydrogenase as a mammalian pyrroloquinoline quinone (PQQ)-binding protein. *Sci. Rep.* **6**, 26723; doi: 10.1038/srep26723 (2016).

## Supplementary Material

Supplementary Information

## Figures and Tables

**Table 1:** List of proteins identified from EAH-Sepharose eluates by nano-LC-MS/MS.

No.	Protein name	GI no.	Score	M.W.	Identified sequence
1	Serum albumin	20330098	165	70,700	CSSMQKFGER (Oxi-M)LGEYGFQNAILVRLGEYGFQNAILVRLGEYGFQNAILVRECCHGDLLECADDR
2	Actin, cytoplasmic 1	6671509	131	42,052	AGFAGDDAPRDLTDYLMKGYSFTTTAEREITALAPSTMKEITALAPSTMK (Oxi-M)AVFPSIVGRPRDSYVGDEAQSKRQEYDESGPSIVHRVAPEEHPVLLTEAPLNPK
3	Vimentin	138536	65	53,712	FANYIDKFADLSEAANREYQDLLNVKILLAELEQLK
4	Glyceraldehyde-3-phosphate dehydrogenase	120702	46	36,072	VGVNGFGRVIPELNGKGAAQNIIPASTGAAKIVSNASCTTNCLAPLAK

**Table 2:** List of proteins identified from PQQ-Sepharose eluates by nano-LC-MS/MS.

**No**	**Protein name**	**GI no.**	**Score**	**M.W.**	**Identified sequence**
1	Pyruvate kinase PKM	146345448	205	58,378	APIIAVTRGIFPVLCKVNLAMDVGKVNLAMDVGK (Oxi-M)GSGTAEVELKGDYPLEAVRGDLGIEIPAEKLDIDSAPITARNTGIICTIGPASRIYVDDGLISLQVKRFDEILEASDGIMVAR (Oxi-M)LNFSHGTHEYHAETIK
2	Nucleoside diphosphate kinase B	117606270	114	17,466	NIIHGSDSVESAEKVMLGETNPADSKPGTIRVMLGETNPADSKPGTIR (Oxi-M)
3	l-Lactate dehydrogenase A chain	126048	113	36,817	LVIITAGARSADTLWGIQKVTLTPEEEARVIGSGCNLDSAR
4	Serum albumin	20330098	106	70,700	LGEYGFQNAILVRLGEYGFQNAILVRLGEYGFQNAILVRECCHGDLLECADDR
5	Actin, cytoplasmic 1	6671509	104	42,052	AGFAGDDAPRDLTDYLMK (Oxi-M)GYSFTTTAEREITALAPSTMKEITALAPSTMK (Oxi-M)AVFPSIVGRPRDSYVGDEAQSKRVAPEEHPVLLTEAPLNPK
6	Glyceraldehyde-3-phosphate dehydrogenase	120702	82	36,072	VGVNGFGRLTGMAFR (Oxi-M)GAAQNIIPASTGAAKVVDLMAYMASKE (Oxi-M)VPTPNVSVVDLTCRIVSNASCTTNCLAPLAK
7	Elongation factor 1-alpha 1	56405010	78	50,424	EVSTYIKQLIVGVNKQTVAVGVIKIGGIGTVPVGREHALLAYTLGVKYYVTIIDAPGHR
8	Peroxiredoxin-1	547923	62	22,390	SVDEIIRADEGISFRGLFIIDDKLVQAFQFTDKQITINDLPVGRGLFIIDDKGILR
9	Triosephosphate isomerase	353526354	49	32,684	VVFEQTKIAVAAQNCYKVIADNVKDWSKIIYGGSVTGATCKHVFGESDELIGQK
