# Supplementary material for: Identification of lactate dehydrogenase as a mammalian pyrroloquinoline quinone (PQQ)-binding protein
Source: Sci Rep. 2016 May 27;6:26723. doi: 10.1038/srep26723 (PMC4882622; doi:10.1038/srep26723)
Supplement: Supplementary Information [file srep26723-s1.pdf]

Supplementary Information

Identification of lactate dehydrogenase as a mammalian pyrroloquinoline quinone (PQQ)-binding protein

Mitsugu Akagawa[1], Kenji Minematsu[1], Takahiro Shibata[2,3], Tatsuhiko Kondo[2], Takeshi Ishii[4] & Koji Uchida[2]

[1] Department of Biological Chemistry, Division of Applied Life Science, Graduate School of Life and Environmental Sciences, Osaka Prefecture University, Sakai 599-8531, Japan

[2] Graduate School of Bioagricultural Sciences, Nagoya University, Nagoya 464-8601, Japan

[3] PRESTO, Japan Science and Technology Agency (JST), Kawaguchi, Saitama 332-0012, Japan

[4] Faculty of Nutrition, Kobe Gakuin University, Kobe, Hyogo 651-8586, Japan

E-mail: akagawa@biochem.osakafu-u.ac.jp (M.A.) or uchidak@agr.nagoya-u.ac.jp (K.U.)

Supplementary Figure S1

```
Mouse    1    ATLKDQLIVNLLKEEQAPQNKITVVGVGAVGMACAISILMKDLADELALVDVMEDKLKG
Rabbit   1    AALKDQLIHNLLKEEHVPQNKITVVGVGAVGMACAISILMKDLADELALVDVMEDKLKG
Human    1    ATLKDQLIYNLLKEEQTPQNKITVVGVGAVGMACAISILMKDLADELALVDVIEDKLKG

Mouse    61   EMMDLQHGSLFLKTPKIVSSKDYCVTANSKLVIITAGARQQEGESRLNLVQRNVNIFKFI
Rabbit   61   EMMDLQHGSLFLRTPKIVSGKDYSVTANSKLVIITAGARQQEGESRLNLVQRNVNIFKFI
Human    61   EMMDLQHGSLFLRTPKIVSGKDYNVTANSKLVIITAGARQQEGESRLNLVQRNVNIFKFI
                                                    Δ

Mouse    121  IPNIVKYSPHCKLLIVSNPVDILTYVAWKISGFPKNRVIGSGCNLDSARFRYLMGERLGV
Rabbit   121  IPNVVKYSPHCKLLVVSNPVDILTYVAWKISGFPKNRVIGSGCNLDSARFRYLMGERLGV
Human    121  IPNVVKYSPNCKLLIVSNPVDILTYVAWKISGFPKNRVIGSGCNLDSARFRYLMGERLGV
                                                              Δ

Mouse    181  HALSCHGWVLGEHGDSSVPVWSGVNVAGVSLKSLNPELGTDADKEQWKEVHKQVVDSAYE
Rabbit   181  HALSCHGWILGEHGDSSVPVWSGMNVAGVSLKTLHPELGTDADKEQWKQVHKQVVDSAYE
Human    181  HPLSCHGWVLGEHGDSSVPVWSGMNVAGVSLKTLHPDLGTDKDKEQWKEVHKQVVESAYE

Mouse    241  VIKLKGYTSWAIGLSVADLAESIMKNLRRVHPISTMIKGLYGINEDVFLSVPCILGQNGIS
Rabbit   241  VIKLKGYTTWAIGLSVADLAESIMKNLRRVHPISTMLKGLYGIKEDVFLSVPCVLGQNGIS
Human    241  VIKLKGYTSWAIGLSVADLAESIMKNLRRVHPVSTMIKGLYGIKDDVFLSVPCILGQNGIS

Mouse    301  DVVKVTLTPEEEARLKKSADTLWGIQKELQF
Rabbit   301  DVVKVTLTSEEEAHLKKSADTLWGIQKELQF
Human    301  DLVKVTLTSEEEARLKKSADTLWGIQKELQF
```

Fig. S1. Alignment of the amino acid sequences of LDH-A of mouse, rabbit, and human[1]. Perfect matches are enclosed in boxes with a black background. The open arrowheads indicate the position of the proposed residues involved in PQQ binding.

Supplementary Figure S2

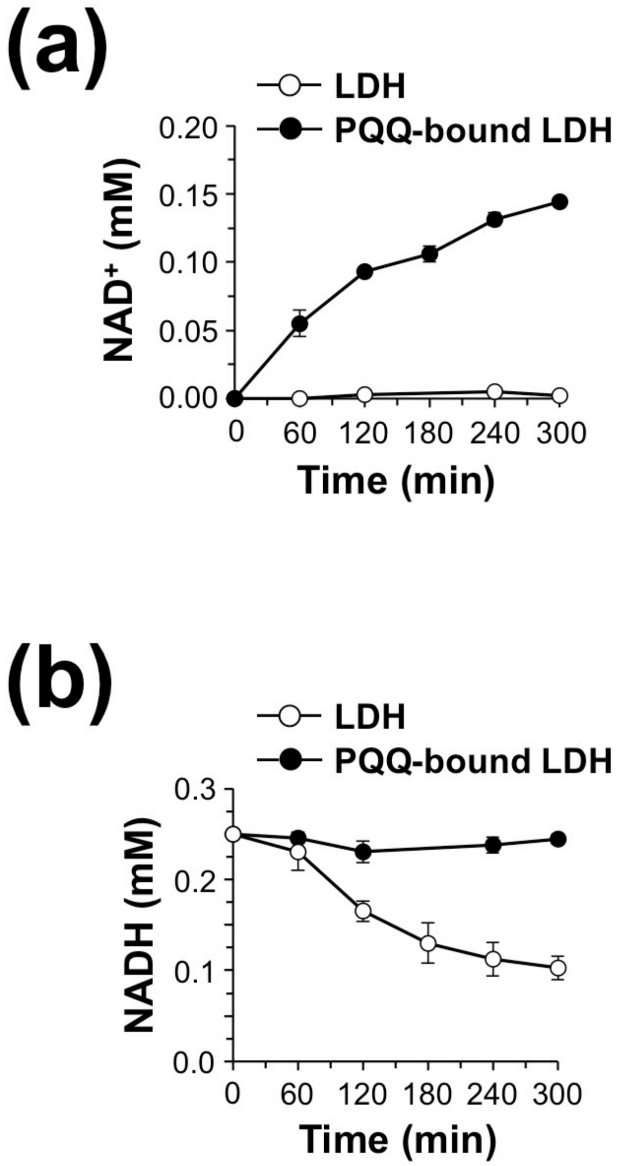

Fig. S2. Time course of NAD^+ formation by PQQ-bound LDH in the presence of NADH. Rabbit muscle LDH (600 nM) and PQQ-bound LDH (600 nM) were incubated with 0.25 mM NADH at 37 ˚C for the indicated time. Then, concentrations of NAD^+ (**a**) and NADH (**b**) in the reaction mixtures were determined by HPLC. The results shown are means ± SE (*n* = 3).
